# Hypoxia and Intestinal Inflammation: Common Molecular Mechanisms and Signaling Pathways

**DOI:** 10.3390/ijms24032425

**Published:** 2023-01-26

**Authors:** Kristina A. Dvornikova, Olga N. Platonova, Elena Y. Bystrova

**Affiliations:** I.P. Pavlov Institute of Physiology RAS, 199034 St. Petersburg, Russia

**Keywords:** hypoxia, HIF-1α, HIF-2α, innate immunity, adaptive immunity, inflammatory bowel disease, ulcerative colitis, Crohn’s disease, inflammation

## Abstract

The gastrointestinal tract (GI) has a unique oxygenation profile. It should be noted that the state of hypoxia can be characteristic of both normal and pathological conditions. Hypoxia-inducible factors (HIF) play a key role in mediating the response to hypoxia, and they are tightly regulated by a group of enzymes called HIF prolyl hydroxylases (PHD). In this review, we discuss the involvement of inflammation hypoxia and signaling pathways in the pathogenesis of inflammatory bowel disease (IBD) and elaborate in detail on the role of HIF in multiple immune reactions during intestinal inflammation. We emphasize the critical influence of tissue microenvironment and highlight the existence of overlapping functions and immune responses mediated by the same molecular mechanisms. Finally, we also provide an update on the development of corresponding therapeutic approaches that would be useful for treatment or prophylaxis of inflammatory bowel disease.

## 1. Introduction

A hallmark of inflamed tissues is the condition hypoxia, which is characterized by vascular dysfunction and increased oxygen consumption due to immune cells infiltration. It is self-evident that hypoxia and inflammation are interconnected at molecular and cellular levels. Both Crohn’s disease (CD) and ulcerative colitis (UC) are chronic inflammatory diseases brought on by dysregulated immune responses to luminal antigens. It is widely recognized that the pathogenesis of inflammatory bowel disease (IBD) is also influenced by genetic and microbiota determinants. Furthermore, intestinal inflammation is linked to mucosal hypoxia according to an increasing number of studies. However, the existence of multiple signaling pathways using the same activating and inhibitory molecules can lead to cross-signaling. For this reason, the available data are often contradictory. On the one hand, a multilevel regulation system makes it possible to identify many targets for the development of therapeutic approaches for the effective treatment of inflammation induced by hypoxia. On the other hand, it requires a comprehensive understanding of the complex molecular mechanisms involved in maintaining gut homeostasis. In this review, we aim to outline the major roles of hypoxia-inducible factor (HIF) in the onset and progression of hypoxia and gut inflammation with a strong emphasis on understanding the complex pathophysiology of the intestine, and to consider in detail possible molecular interactions between HIF and innate/adaptive immunity under normal and pathological (IBD) conditions.

## 2. Hypoxia and the Gastrointestinal Tract

### 2.1. Role of Oxygen in the GI and Adaptation to Hypoxia

The intestinal microbiota in the intestinal lumen and the oxygen metabolism of intestinal epithelial cells (IECs) during food digestion and nutrient absorption both contribute to the particular oxygenation profile of the gastrointestinal tract (GI) [1]. The dynamic and quick changes of oxygen tension in cells maintain oxygen homeostasis in a healthy gut. A decrease in oxygen levels of longitudinal and radial steep spatial oxygen gradients, which occur in the longitudinal direction from the small to the large intestine and in the radial direction from the submucosal plexus into the intestinal lumen, are intrinsic features of the GI. In the physiological state, the mucous membrane of the intestine and stomach is affected by partial pressure O2 (pO2) [2]. Therefore, pO2 in the stomach is 58 mm Hg, in the duodenum 32 mm Hg, in the ascending colon 11 mm Hg, and in the sigmoid colon 3 mm Hg. In contrast, pO2 in the alveoli of healthy lungs ranges from 100 to 110 mm Hg, and pO2 in the air we breathe is 145 mmHg. In addition, a newborn’s intestine is better oxygenated as compared to an adult’s GI [3].

The normal functioning of the digestive tract is significantly influenced by the intestinal microbiota. In particular, it is involved in the breakdown of food nutrients, upkeep of intestinal barrier integrity, and control of systemic immunological responses [4]. The microbiota is likewise essential in developing a hypoxic intestine microenvironment, because the intestinal microbiome of an adult is composed overwhelmingly of anaerobes that lessen oxygen content [5]. At the same time, facultative anaerobes consume available oxygen, create an anaerobic microenvironment withinside the gut and make contributions to colonization of the gut by obligate anaerobes. In particular, patients with celiac disease have more potentially pathogenic gut bacteria and fewer beneficial species compared to healthy controls. Gluten-induced dysbiosis results in tight-junction barrier dysfunction and activation of certain inflammatory pathways. It has been further shown that application of gliadins, which are a component of gluten, can suppress growth and morphological alterations in multiple cell lines of human origin and induce oxidative stress, thus allowing speculation on a possible crosstalk between gluten-associated dysbiosis and hypoxia [6]. It is noted that antibiotics use reducing the species, and quantitative make-up of the microbiota cause the increase of oxygen level and proliferation of aerobic bacteria that can subsequently result in a pathological state and epithelial damage [7]. In view of the above, we can speculate that the gut functioning highly depends on a number of adaptive pathways associated with hypoxia.

Adaptation to hypoxia at the cellular level in the gut in one aspect is regulated by hypoxia-inducible factors (HIF). HIF are heterodimeric transcription factors consisting of oxygen-unstable α-subunits (HIF-1α, HIF-2α, HIF-3α) and a constitutively expressed β-subunit (HIF-1β, also known as aryl hydrocarbon receptor nuclear translocator (ARNT)), which, inter alia, are involved in activation of innate and adaptive immunity [8]. It has been established that HIF-1α and HIF-2α are expressed in IECs [9,10]. HIF activate transcription by binding to hypoxia response elements (HRE) present in a wide range of genes, thus regulating the cellular response to hypoxia [11]. HIF-α stabilizes and forms a heterodimer with the HIF-β subunit. The HIF-α subunit comprises a highly conserved oxygen-dependent degradation (ODD) domain. The main oxygen sensors in the cell are enzymes of the prolyl hydroxylase domain (PHD)—α-ketoglutarate-dependent dioxygenases, consisting of PHD1 (EGLN1), PHD2 (EGLN2), and PHD3 (EGLN3) isoforms. It is reported that PHD2 and PHD3 mRNA levels increase as a result of hypoxia, and PHD1 is not regulated by such feedback mechanism [12]. Under conditions of sufficient oxygen content, HIF is hydroxylated by PHD enzymes, which leads to its ubiquitination by Von Hippel–Lindau (VHL) tumor suppressor protein containing E3-ubiquitin ligase complex known as ECV (Elongins B/C, CUL2, VHL), and subsequent degradation and decrease to low basal levels in normoxia [13,14]. Oxygen-dependent asparagine hydroxylase—a factor inhibiting HIF (FIH)—also plays an important role since it is capable of inhibiting HIF-α activity in normoxia due to degradation and transcription repression [15].

### 2.2. Gut Metabolites and HIF Activity

Metabolites that can alter the stability of HIF-α are important for cellular metabolism. Thus, α-ketoglutarate is required for production of succinyl-CoA, an intermediate of tricarboxylic acid cycle (TCA cycle) in IECs. Mutations in α-ketoglutarate-producing enzymes (isocitrate dehydrogenase 1 (IDH1) and isocitrate dehydrogenase 2 (IDH2)) lead to a decrease in isocitrate/α-ketoglutarate conversion and an increase in the activity of the neomorphic IDH1 enzyme, thus resulting in growth of 2-hydroxyglutarate level being a competitive inhibitor of α-ketoglutarate, which reduces PHD activity [16]. Another metabolite, succinate, is formed after HIF-1α hydroxylation, can directly inhibit PHD activity, and furthermore induce interleukin-1β (IL-1β) production in macrophages via HIF-1α-associated signaling pathways [17]. Thus, it is clearly evident that metabolites, in addition to their classical function as intermediates in the metabolic pathway, are not only able to modulate HIF-1α activation but can also direct immune responses. This fact represents an important turning point for immunology since the role of metabolites is expanding to include mediation of immune responses.

In turn, PHD enzymatic activity requires Fe^2+^, an important trace element for proper oxygen transport, as a cofactor [18]. The development of hypoxia and signaling pathways involving Fe^2+^ are closely connected. Fe^2+^ can be transported via divalent metal transporter-1 (DMT1/NRAMP2). At that, HIF-2α is required to uptake Fe^2+^ by regulating the expression of duodenal ferric reductase (DcytB), DMT1, and basolateral iron transporter ferroportin (FPN, also known as *Slc40a1*) in the gut and maintaining the integrity of the epithelial barrier. Accordingly, following Fe^2+^ deficiency, PHD activity decreases, which results in HIF-2α activation. In this case, HIF-2α can directly regulate the expression of DcytB, DMT1, and FPN.

Hepcidin, a hepatic peptide hormone that regulates Fe^2+^ absorption in the intestine, can bind to FPN [19]. At that, an increase in hepcidin expression leads to FPN degradation and a decrease in Fe^2+^ transport into plasma, while a decrease in hepcidin expression vice versa results in a high outflow of Fe^2+^ from the intestinal epithelium. Hepcidin has also been found to regulate intestinal HIF-2α in Fe^2+^ deficiency, anemia, and Fe^2+^ overload [19]. Therefore, low hepcidin levels may increase corresponding HIF-2α levels by decreasing PHD activity.

In addition, mitochondrial reactive oxygen species (ROS) are significant for HIF activation. It has been established that hypoxia increases generation of ROS in the mitochondrial complex III, adding to the accumulation of HIF-1α protein. This proves the importance of mitochondrial ROS for initiating HIF-1α stabilization during hypoxia [20]. However, the exact mechanism of said ROS-mediated stabilization of HIF-1α is unknown.

Diet also affects HIF activity [21]. Intestinal HIF-2α signaling, but not HIF-1α, has been shown to be activated in obesity. There is a positive correlation between HIF-2α signaling in the gut and body mass index, as well as a high-fat diet [22]. Thus, mice with obesity induced by a high-fat diet and *Hif2a* gene transcriptional activity inhibition were characterized by less severe forms of hepatic steatosis as compared to healthy control animals. Intestinal HIF-2α regulates ceramide metabolism, and inhibition of HIF-2α markedly decreases intestinal and serum ceramide levels. Therefore, it is suggested that intestinal HIF-2α plays a central role in the regulation of obesity and steatosis and can be considered as a target for treatment of hepatic steatosis.

The activity of HIF is also affected by physiological gaseous signaling mediators (gas transmitters) that interact with HIF signaling pathways and can modulate them [13]. Thus, nitric oxide (NO), hydrogen sulfide (H_2_S), and carbon dioxide (CO_2_) inhibit HIF, while carbon monoxide (CO) and ammonia (NH_3_) activate it [23]. It should be noted that gas transmitters content is altered in an inflammatory state, which means that their levels will modulate the level of HIF activity. For example, NO production increases during inflammation and inhibits HIF hydroxylases, resulting in increased HIF activity, wherein low NO concentrations inhibit mitochondrial oxygen uptake [24]. Depending on the pathological condition and the level of gas transmitters, not only changes in the stability of HIF will take place but also metabolic and effector consequences in the immune cells. Therefore, it is important to take a closer look at the relationship between gut immunity and HIF.

### 2.3. Gut Immunity and HIF

Oxygen is a central element for tissue oxidative metabolism, and understanding of cellular and tissue responses to hypoxia can provide essential information about immune function [25]. At that, it is important to differentiate between physiological hypoxia that takes place in healthy tissue and inflammatory hypoxia induced by active inflammation. In both cases, changes in tissue oxygenation will lead to active recruitment of immune cells of innate and adaptive immunity, such as neutrophils, eosinophils, macrophages, innate lymphoid cells (ILCs), as well as to local proliferation of T- and B-cells [26]. Consequently, it is important to understand the crossing of metabolism with active immunity in the local tissue environment. It has been established that the cells of the innate and adaptive immunity are subjected to hypoxia, and HIF affects their gene expression and subsequent effector function [27]. One of the suggested mechanisms is the control of cellular metabolism, which allows HIF to regulate the function of the immune cells [28]. In particular, HIF-1α increases glycolysis rate due to transcriptional regulation of genes encoding glycolytic enzymes—aldolase A (ALDA), phosphoglycerate kinase 1 (PGK1), and pyruvate kinase M. In turn, an increased rate of glycolysis is associated with the activation of macrophages, dendritic cells (DCs), and T- and B-cells. Accordingly, it can be suggested that HIF is an important regulator of innate and adaptive immunity. It should also be noted that HIF-1α is expressed in all types of immune cells, HIF-2α is more selectively expressed in neutrophils, natural killer (NK) cells associated with tumors, macrophages, and activated T-cells under hypoxic conditions [26].

IECs originate from intestinal stem cells (ISCs) located at the bottom of the intestinal crypts, which are constantly proliferating and differentiating into mature cells [29]. In fact, HIF pathways are involved in regulation of ISC self-renewal and differentiation into various other intestinal cells (e.g., enteroendocrine and Paneth cells). Recent studies suggest that HIF shapes normal intestinal metabolism and is central to the coordination of barrier regulation during both homeostasis and active disease [30]. In acute inflammation, HIF is able to control the rapid restitution of the epithelium. At the same time, HIF may contribute to the fibrosis associated with chronic inflammation. It is assumed that the role of HIF may be different depending on the context of inflammation.

Immune responses in the gut are substantially coordinated by IECs and intestinal intraepithelial lymphocytes (IELs) [31]. IELs are located between IECs and are the first cells to come into contact with luminal antigens that penetrate the mucosa [32]. Most IELs are CD8+ T-lymphocytes, which play a critical role in maintaining intestinal homeostasis. HIF-1α derived from IECs is reported to be required for supporting homeostasis of IELs and gut microbiota by functionally altering CD8αα+ IELs during proliferation, apoptosis, and immigration [10]. However, the existing interactions between HIF-1α, CD8αα+ IELs, and the microbiome are still to be brought to light. It has been shown that stabilization of HIF-1α at low oxygen levels triggers the expression of genes that increase IECs’ abilities to function effectively as a barrier [26]. IECs are covered with mucus, which is an important barrier to pathogens. Mucus is composed of a number of secreted mucin and stabilizing proteins, including intestinal trefoil factor (ITF) [33]. Cells present in the intestinal mucosa produce antimicrobial peptides (AMPs)—α-defensins, β-defensin-1, lysozyme, secretory phospholipase 2, angiogenin 4, lectin, and cathelicidins—important endogenously produced factors that have antimicrobial properties and may modulate the function of innate immune cells [34]. At that, HIF contributes to regulation of gene expression responsible for the production and stability of mucus (MUC3 and ITF) and AMP synthesis, as well as maintenance of the intestinal barrier function under hypoxic conditions [35,36]. HIF affects barrier function of IECs directly through regulation of an epithelial tight junction (TJ) component [37]. In particular, claudin-1 (CLDN1) has been reported as a transcriptional target of HIF [37]. Further, the central role for HIF signaling in junction integrity and homeostasis has been suggested. Knockdown of *HIF-1β* in vitro resulted in significant defects in barrier function, as well as in severe morphological abnormalities in the architecture of the tight junction. Thus, HIF is able to mediate barrier function and provides a new mechanism of the profound barrier-protective effects associated with HIF signaling.

In addition, HIF-1α regulates gene expression in autophagy and xenophagy, associated with mucosal immunity in the gut [38]. Gene variants that regulate autophagy are genes of high risk for inflammatory bowel disease and include autophagy-related 16-like (ATG16L1) and immunity-related GTPase family M (IRGM). It is known that the mucosal surface, as a result of microbiota metabolism, provides synthesis of a number of signaling molecules, for example, short-chain fatty acids (SCFAs), including butyrate, propionate, and acetate [39]. Said SCFAs (in particular, butyrate) can induce autophagy in IECs, stimulate epithelial metabolism, alter gene expression, and increase epithelial oxygen consumption, which, in turn, leads to stabilization of HIF transcription factor [40]. At that, in a dextran sodium sulfate (DSS) model of colitis, administration of butyrate facilitated colon damage and suppressed inflammation in Cre-/HIF-1α- mice. However, butyrate-mediated protection against colon injury was significantly reduced in HIF-1α∆IEC mice. These results demonstrate that, taken together, HIF-1α, autophagy, and gut microbiota are factors required for maintenance of gut homeostasis. In addition, a bidirectional relationship between autophagy and HIF-1α should be noted. However, the exact mechanisms of butyrate-mediated autophagy induction are still to be brought to light.

Thus, we can hypothesize that hypoxia and HIF/PHD activity have profound effects on the cells of the innate and adaptive immune system of the gut.

#### 2.3.1. Innate Immunity of the Intestine and HIF

Innate immunity cells express various pattern recognition receptors (PRRs), the key ones being Toll-like receptors (TLRs). TLRs recognize pathogen-associated molecular patterns (PAMPs) originating from microorganisms or representing endogenous molecules, which result in activation of the innate immune response [41]. TLRs play an important role in maintaining intestinal homeostasis, maturation of DCs, and induction of proliferation and differentiation of T helper 1 (Th1) and T helper 2 (Th2) [42]. In NK cells, HIF-2α limits cellular cytotoxicity, which may indicate its potential anti-inflammatory role [43].

There is evidence that HIF-1α and HIF-2α are involved in the control of motility, bactericidal activity, and oncogenic potential of macrophages [44]. Activation of HIF may promote polarization of macrophages. It is known that HIF-1α-dependent glycolysis mediates polarization of macrophages through M1 line, and polarization through M2 line depends on HIF-2α [45]. Said polarization results in changes associated with the production and metabolism of inflammatory cytokines. In addition, metabolites and metabolic enzymes—succinate, glycolytic regulator pyruvate dehydrogenase kinase 1 (PDK1), and the key regulator of pyruvate kinase M2 (PKM2) metabolism—may also play a role in HIF-1α-dependent M1 polarization [46]. Recently, it has been shown that lipopolysaccharide (LPS), a TLR4 ligand, is able to activate HIF-1α expression in macrophages [12]. These results also demonstrate that LPS exposure leads to a shift in M2 with increased IL-10 production and reduced inflammation.

DCs, along with macrophages, serve as a bridge between innate and adaptive immunity [47]. Activation of the HIF-1α signaling pathway has been shown to modulate several functions of DCs, including survival, differentiation, maturation, migration, and antigen presentation [48]. It has also been demonstrated that HIF-1α is an important regulator of IFN-γ synthesis, and interleukin-22 (IL-22) and interleukin-10 (IL-10) production in DCs. Additionally, hypoxia increases the expression of neutrophil-attracting chemokines, such as CXCR2, CXCR3, CCR5, and CXCL8 [49]. Neutrophils induce the elimination of pathogens through ROS, NO, and phagocytosis, while HIF-1α signaling and expression of PHD3 enzymes determine the long-term survival of neutrophils under hypoxic conditions [50]. Neutrophil activation and migration rapidly deplete local oxygen and lead to localized induction of HIF signaling in IECs, increasing barrier function and preventing further infiltration of pro-inflammatory immune cells [51].

It has been established that stabilization of HIF-1α plays a crucial role in maintaining the survival and functioning of basophils, eosinophils, and mast cells, and activation of HIF-1α signaling pathway is necessary to control interleukin-8 (IL-8) and tumor necrosis factor-α (TNF-α) synthesis by mast cells after stimulation with TLR ligand [26]. HIF-1α is important in supporting IgE-mediated basophil inflammatory responses by regulating the release of interleukin-4 (IL-4) and proangiogenic VEGF, and it is also suggested that HIF-1α maintains basophil activity during prolonged inflammation [52]. However, studies of mast cells, basophils, and eosinophils in the context of their relationship with HIF are insufficient to form a clear understanding of how their function and metabolism are modulated by hypoxia and the HIF signaling pathway.

Furthermore, there is a lack of studies regarding association of HIF with ILCs. It is known that ILCs provide innate immunity to maintain the intestinal barrier, mediating a rapid response to pathogens and secreting a large number of cytokines [53]. A conditional deletion of VHL ligase E3 component has been shown to result in a selective defect in the function of mature ILC2 and a decrease in the type 2 immune response [54]. VHL deficiency-induced accumulation of HIF-1α and attenuated interleukin-33 (IL-33) receptor ST2 expression, which could be avoided by removing or inhibiting HIF-1α. Functional defects have been associated with HIF-1 stabilization and increased glycolysis. Further research of HIF-ILC interconnection will provide a new understanding of the innate immunity role in HIF-1α-dependent pathways.

#### 2.3.2. Adaptive Immunity of the Intestine and HIF

There is confident evidence that HIF is involved in the development and functioning of T- and B-cells [55]. T-cells are exposed to hypoxia during their recruitment from inflamed regions to secondary lymphoid tissues such as spleen and mesenteric lymph nodes. Activation of HIF-1α is known to promote a metabolic shift towards glycolysis as well as alteration of transcriptional responses in differentiating T-cells by regulating the retinoic acid-associated orphan receptor γt (RORγt, also known as RORγ2) and the transcription factor Tregs forkhead box P3 (FoxP3), creating an environment favorable for differentiation into Th17 [56]. At that, in activated T-cells, HIF-1α mediates cytolytic, migratory, and co-stimulating properties, however, it is important to take into account the negative HIF-1α regulation of Th1 cell function, depending on the conditions and context. In particular, Th1 loses the ability to produce IFN-γ when cultivated under hypoxic conditions [57]. Another study has reported that HIF-1α-driven FoxP3 is required for implementing functions by regulatory T-cells (Tregs), wherein cells lacking HIF-1α have reduced anti-inflammatory capacity and lose the ability to control inflammation [58]. At the same time, the depletion of VHL in Tregs results in HIF-1α activation and transformation of Tregs into Th1, thereby contributing to the development of inflammation [59].

It has been previously demonstrated that, following in vitro stimulation, Th1, Th2, and Th17 utilize glycolytic metabolism, in contrast to in vitro-activated Tregs, which exhibit increased lipid oxidation and oxidative phosphorylation [60]. Activation of HIF-1α in CD8+ T-cells has been shown to promote glycolytic metabolism, which is required for effector function, while hypoxia and the HIF signaling pathway affect tumor infiltration by cytotoxic T-cells [61]. Therefore, it can be concluded that HIF-1α plays a central role in T-cell differentiation by stimulating glycolytic metabolism and controlling main aspects of T-cell functioning. Accordingly, HIF-1α can be considered as a key component of adaptive immunity mediated by T-cells.

HIF-1α plays an important role in the expression of the alkaline pH-activated two-pore domain K+ channel K2P5.1 (TASK-2, also known as KCNK5) in B-cells, which is required to maintain B-cell functions such as proliferation, survival, or production of cytokines [62]. HIF also increases glycolytic metabolism in B-cells of a germinal center (GC), which affects B-cell proliferation, apoptosis, and antibody production [63]. It is suggested that, due to modulation of glycolytic metabolism, HIF-1α affects a specific population of CD1dhiCD5+ B-cells, directly controlling IL-10 production [64]. As a consequence, the expression of HIF-1α in B-cells regulates inflammatory conditions, thus providing a possible approach for increasing the immunoregulatory potential of B-cells producing IL-10, as an option for preventing and/or treating inflammatory diseases. At that, it is known that PHD inhibitors increase the number of B-cells producing IL-10 and reduce the expression of inflammatory cytokines [65]. Since HIF is hydroxylated by PHD enzymes, which, in particular, decreases HIF levels in normoxia, it can be expected that exposure to PHD inhibitors increases HIF levels and, as a result, leads to increased production of IL-10, preventing intestinal inflammation.

Thus, understanding the role of HIF in the functioning of B-cells will make it possible to use the obtained results in the field of therapeutic modulation of immune responses for treatment of inflammatory diseases.

Nuclear factor κB (NF-κB) is a hypoxia-sensitive transcription factor that plays an essential role in control of the inflammatory response. Activation of NF-κB can enhance HIF response under conditions of hypoxic inflammation [66]. Hypoxia or inhibition of PHD induces an increase in NF-kB activity in some cell types, or suppression of NF-kB activity in cells after exposure to IL-1β or LPS [67]. This reflects the complex regulatory role of hypoxia in relation to the NF-κB pathway. HIF-1β has also been reported to regulate NF-κB activity through direct control of TRAF6, an adapter protein capable of connecting signaling pathways associated with TNFR and IL-1β/TLRs with implications for homeostasis and inflammation [68]. As a result, HIF-1β-mediated control of TRAF6 appears to be necessary for full NF-κB activity in cells, and this regulatory mechanism controls cell survival. The identified association between HIF-1β and NF-κB could be essential for signaling in inflammatory diseases. Further studies will allow identifying exact mechanisms of direct regulation. According to the available data, it can be concluded that NF-κB is the second hypoxia-sensitive transcription factor that plays a key role in the regulation of inflammation and immunity due to its important role in the control of immune cells function. At that, NF-κB can induce a pro- or anti-inflammatory response, depending on the cell type, level, and duration of its activity in the context of a particular inflammation.

It should be noted that HIF’s role in immune cells is not unambiguous. Indeed, the HIF signaling pathway modulates most cellular processes, but there are several levels of regulation, including positive and negative feedback, and cross-signaling. As mentioned previously, there are important differences in the regulation of HIF by hypoxia compared to the regulation by inflammatory stimuli such as LPS. HIF is required for later transition to glycolysis that occurs in DCs, but the immediate metabolic switch is not dependent on HIF and is mediated by an important intracellular signaling pathway ROS/PI3K (phosphatidylinositol 3-kinases)/Akt (Protein kinase B (PKB)) in metabolism regulation [69]. Many issues still need clarification and further investigation.

## 3. Hypoxia and IBD

### 3.1. General Information on IBD

Crohn’s disease (CD), ulcerative colitis (UC), microscopic colitis (MC), and the less common inflammatory bowel disease unclassified (IBDU), which includes variations with an unclear clinical pattern, are all chronic inflammatory disorders of the gastrointestinal tract that fall under the general term, “inflammatory bowel disease” (IBD) [70,71]. In Europe, the prevalence of IBD is the highest, at 505/1,000,000 for UC and 322/100,000 for CD. The number of new cases has also been increasing dramatically in developing countries. According to estimates based on the Swedish population, MC affects 1 in 115 women and 1 in 286 men during their lifetime, contrary to earlier beliefs that it was a rare cause of chronic watery diarrhea in high-income countries [71]. IBD multifaceted etiology and the complex regulation of persistent immune responses that come from the combination of genetic, environmental, and immunological variables mean that the problem is still difficult to solve [72].

The gut mucosa is the first and most crucial part of the defense line when it comes to bowel disease [73]. The degree to which environmental antigens penetrate the intestinal wall and how far they are presented to immune system cells depends on the integrity of the intestinal barrier. The epithelial cells connected by efficient cell junctions, the mucosal layer that covers them, and the unique microbiome pattern are the key components of the boundary. The predominance of pathogenic strains, coming from overexposure to antibiotics and industrial chemicals, contributes to the increase in the incidence of IBD. It is also known that disruption of microbiota (dysbiosis) is a cause of the exacerbation of the disease and determines the severity of the inflammation [74].

Some pathogen types are more frequently associated with a higher risk of the occurrence and progression of IBD, despite the highly personalized nature of the microbiome. By releasing Toxin A and Toxin B, which bind to epithelial cells, *Clostridium difficile* triggers the production of proinflammatory cytokines such as TNF-α and IL-6, IL-8, and interleukin-1 (IL-1), which disrupt the integrity of the intestinal barrier [75]. In addition, a number of bacteria, such as enteroinvasive and enteroadherent serotypes of *Escherichia coli*, *Campylobacter jejuni*, *Salmonella typhi*, *Mycobacterium avium*, and *Mycobacterium paratuberculosis*, are able to also be involved in the pathogenesis of IBD [76]. For example, UC-associated *E. coli* producing α-hemolysin has been shown to promote a rapid loss of tight junction integrity on the model of differentiated monolayers of Caco-2 cells, thus suggesting a high level of α-hemolysin expression to be a possible mechanism involved in epithelial barrier dysfunction and IBD progression [77]. Another study implicates that *Mycobacterium avium* subspecies *paratuberculosis* is associated with CD onset in genetically predisposed patients (e.g., having mutations in *TLR4* and *IL10RA* genes), being a specific trigger in CD pathogenesis [78]. Furthermore, it is known that gut microbiota plays an important role by maintaining intestinal homeostasis, providing assimilation of diet nutrients, synthetizing a number of SCFAs, vitamins, and essential amino acids, and supporting the immune microenvironment of the intestine, thus mediating the prevention/management of pathological conditions, inter alia, inflammation in IBD. Consequently, it is not surprising that IBD patients are characterized by a reduced diversity/impaired makeup of intestinal bacteria compared to healthy controls. E.g., a significant decrease of taxons *Firmicutes*, *Enterobacteriaceae*, *Bacteroidales*, as well as *Clostridiales*, bacteria producing butyrate, in fecal samples from patients with IBD, has been reported [79,80]. Since butyrate is known to enhance the mucosal barrier function by inducing the production of mucin and antimicrobial peptides, its lack can potentially result in increased inflammation. Indirect evidence for the importance of the microbiota has also been obtained from studies on the treatment of inflammatory bowel disease with antibiotics and diets [81,82,83]. The imbalance in the integrity of the epithelial barrier in relation to the infection appears to be undisputed, even though it is difficult to determine whether inflamed mucosa supports the infection or whether pathogens initially cause the inflammatory response.

Numerous studies on the protective role of the intestinal mucosa have been conducted in recent years, and they have contributed to the identification of specific genetic markers that are essential for the progression of IBD. There is evidence that *C4B* and *NOX1* genes are linked to early-life morbidity. A rise in complement activity toward the microbiome and an intensification of dysbiosis were both positively correlated with the number of *C4B* gene copies. Mutation of the *NOX* gene encoding the catalytic subunit of the nicotinamide adenine dinucleotide phosphate oxidase complex Nox1 (NADPH 1) is responsible for rupture of phagocytes and alters the interface between luminal microbes and the epithelium [84,85].

The integrity of the intestinal barrier is ensured by the recruitment of the desmosomal junction protein desmoglein 2 (DSG2), which is regulated by the glial cell line neurotrophic factor acting through the cAMP and p38 MAPK pathways [86,87]. Goblet cells produce mucins, which are structural components of the intestinal mucosa and mediate interactions between bacteria and the host organism. MUC2 and MUC5AC constitute most of the intestinal mucosal coat. The additional components of this structure include the Fc Gamma Binding Protein (FCGBP), Zymogen Granule Protein 16 (ZG16), Anterior Gradient 2 (AGR2), Chloride Channel Accessory 1 (CLCA1), and Trefoil Factor 3 (TFF3). Transmembrane mucins (MUC 17), which function as messengers between luminal antigens and epithelial cells in response to bacterial stimulation, also seem to be involved in a gate-keeping role [88,89].

The signal arising from the lumen leads further through receptors on effector cells when the epithelial defense barrier is impaired. The cytokine cascade of the cellular immune response and tissue remodeling is affected by triggering membrane TLRs and intracellular NOD receptors in immune cells, including antigen-presenting cells (APCs). A genetically-induced impairment of antigen identification by immune cells associated with a *NOD2* mutation has been described in CD and was the first disease-associated mutation to be discovered. Multiple protective mechanisms, including the activation of autophagy, the stimulation of Paneth cells to create antimicrobial peptides, and the induction of a specific immune response by dendritic cells, are carried out by *NOD2* gene products [90]. The presence of the mutations *R702W, G908R*, and *L1007fsinsC* L1007fs in this gene has also been associated to a rise in the frequency of Crohn’s disease [91].

Through the production of pro- and anti-inflammatory cytokines, the adaptive immune response is controlled by a multilevel regulated network. Th17 lymphocytes are now thought to be more important than T-helper lymphocytes types 1 and 2 in the onset of IBD. It has been demonstrated that, whereas Th2-lymphocyte activity rises in UC by release of IL-4, IL-5, and IL-13, Th1-lymphocyte activity increases in CD through the production of gamma-interferon (IFN-γ) and IL-2, respectively. Th17 activity is regulated through IL-23 (activation) and IL-10 (inhibition), and these lymphocytes are the source of several pro-inflammatory cytokines: IL-17A, IL-17F, IL-22, IL-26, and the chemokine CCL20. By releasing IL-10, monocytic and regulatory T-cells can suppress Th17. Cytokine signaling is mediated through the JAK-STAT pathway [92,93]. 

It is reported that the level of IL-1β increases in active forms of IBD and changes the expression and distribution of tight junction proteins, leading to disruption of epithelial barrier integrity [94]. TLR4 also draws attention in the context of considered issues. TLR4 is known to be important for induction of an inflammatory response, as it provides protection against pathogen entry and maintains mucosal integrity. A multidirectional role of the TLR4 signaling pathway in IBD has been established depending on the stimulus. Thus, inhibition of TLR4 has been acknowledged as an effective target for IBD treatment [95]. Targeting TLR4 synergistically with the pyrine domain-containing receptor (NLRP3), known to be a component of the inflammasome, a multi-protein signaling complex responsible for caspase-1 activation and subsequent maturation of IL-1β and pro-IL-18, is also an effective treatment for IBD. In particular, TLR4 regulates NF-κB p65 activation, which affects the expression of NLRP3/IL-1β [96]. In addition, stimulated intestinal glial cells release NO through the TLR4 pathway, which results in pro-inflammatory cytokine production that exacerbates gut inflammation in UC [97].

A thorough examination of the multiple parts involved in IBD etiology permits the creation of novel therapeutic strategies. These may include inhibition of TNF-α with therapeutic monoclonal antibodies or a combination of antibodies with multiple targets, such as anti-TNF-α and anti-IL12/23. Currently, in the treatment of IBD, Janus kinase inhibitors and antibodies suppressing the migration of leukocytes into the GI are effectively used [72].

### 3.2. Interplay between Hypoxia and Inflammation

Signaling pathways associated with hypoxia are active in almost all cells, including immune and epithelial cells. This is important when considering the effect of hypoxia on inflammation, and especially is relevant for the intestine, which is subjected to local hypoxia and may also be an inflammation site. Hypoxia can take place in the normal physiological state of the intestine (physiological hypoxia), or can develop as a result of impairment (inflammatory hypoxia)—subsequently, it exacerbates inflammation by activating associated signal pathways and affecting the function of immune cells [98]. At that, the function and metabolism of immune cells change greatly as they migrate from the oxygen-rich vasculature to hypoxically-inflamed sites [26]. Practically every inflamed tissue is characterized by hypoxia due to vascular dysfunction and increased oxygen consumption by infiltrating immune cells. These infiltrating immune cells are a key component of any inflammatory response, and their behavior determines the severity and duration of the immune response. Inflammatory hypoxia is provided by a combination of recruited neutrophils (referred to as polymorphonuclear leukocytes (PMN)), eosinophils, and monocytes, as well as a high rate of oxidative metabolism and activation of multiple oxygen-consuming enzymes during inflammation [26]. 

Since the majority of inflammatory cells are recruited to inflammatory lesions, innate immune cells have the potential to essentially enhance current metabolism. In particular, infiltrating neutrophils and eosinophils consume large amounts of oxygen during the oxidative burst, which results in mucosal hypoxia [51]. This is due to the high induced activity of neutrophil NADPH oxidase. In turn, the neutrophil NADPH oxidase complex is responsible for the formation of ROS and is used by innate immune cells (especially PMN) to eliminate invading pathogens. It should be noted that the intestinal mucosa is in a state of constant controlled inflammation due to exposure to lumen antigens. Accordingly, the mucosa represents an immunological niche in which immune cells are exposed to a combination of hypoxia and immune stimuli even in a non-inflamed state. However, inflammation brings a dramatic increase in severity of hypoxia in the intestinal mucosa [99]. The longer inflammation lasts, the more likely hypoxia will turn into a chronic pathological form, which is closely associated with chronic intestinal damage [98]. At the same time, tissue hypoxia in chronically inflamed tissues can be pro-inflammatory and anti-inflammatory, depending on the cell type. Thus, hypoxia in IECs leads to barrier protection via regulation of *MUC3* and *ITF* genes, which were discussed earlier, and in immune cells of the intestinal mucosa hypoxia can promote their activity and survival [35,36].

It can be concluded that hypoxia and inflammation are interconnected at molecular and cellular levels. Obviously, hypoxia plays a complex and ambiguous role in inflammation. In one case, there may be a shift in tissue oxygenation towards inflammatory hypoxia, aggravation of damage, and induction of pro-inflammatory cytokines; alternatively, an anti-inflammatory effect and formation of a stable barrier protection is possible.

### 3.3. Inflammatory Hypoxia in IBD and Implications for the Gut

Intestinal inflammation in IBD is characterized by severe mucosal surface hypoxia and concomitant HIF stabilization [100]. The stabilization of HIF in IBD is caused by changes in the ratio of metabolic supply and consumption, resulting in inflammatory hypoxia. It is important to note that HIF-1α and HIF-2α, as well as PHD isoforms, have different roles in intestinal inflammation and immune response [101].

#### 3.3.1. Molecular Interactions of HIF-1α in IBD

It has been established that HIF-1α plays an important role in IBD pathogenesis, affecting innate and adaptive intestinal immunity [102]. Thus, the general activation of HIF-1α contributes to polarization of Th17 by increasing the expression of RORγt through the activity of STAT3 and the suppression of FoxP3 [56]. At that, as it is known, Th17, along with Th1, are important in the pathogenesis of CD, secreting pro-inflammatory cytokines IL-17, IFN-γ, TNF-α, and TNF-β, which promote inflammation by stimulating the production of IL-1, IL-6, IL-8, IL-12, and IL-18 by macrophages [103]. Therefore, HIF-1α, in the context of Th17 polarization, is required to maintain the ability to control inflammation. Effector T-cell activity is regulated by Tregs, a suppressive subset of CD4+ T-cells that play a role in maintaining immune homeostasis in the gut. However, it is known that mucosal effector T-cells in IBD may be resistant or less sensitive to Tregs-mediated inhibition. In turn, the production of IL-10 by Tregs may be crucial for preventing intestinal inflammation, since Tregs have an anti-inflammatory effect that suppresses subpopulations of effector T-cells [104]. Accordingly, it is hypothesized that HIF-1α-induced impairment of Tregs development may exacerbate IBD progression. In addition, HIF-1α activity enhances glycolysis and glucose uptake, promoting pro-inflammatory responses.

In another aspect, HIF-1α also activates CD8+ cytotoxic T-cells, resulting in increased antiviral and antitumor activity and expression of costimulatory/inhibitory molecules CD137 (4-1BB), Cytotoxic T-lymphocyte antigen-4 (CTLA-4), and glucocorticoid-induced tumor necrosis factor family receptor (GITR) [105]. In addition, HIF-1α promotes functioning of the epithelial barrier in the gut, making HIF a therapeutic target for treatment of IBD associated with epithelial barrier dysfunction. Thus, HIF-1α-dependent IL-33 in IECs acts as a regulatory cytokine in the inflamed mucosa and regulates intestinal inflammation by maintaining mucosal homeostasis [106]. In intestinal inflammation, TNF produced by mucosal immune cells induces HIF-1α expression in IECs by activating NF-kB. HIF-1α then forms a heterodimer with HIF-1β and binds to the IL-33 promoter, resulting in overexpression of IL-33 in IECs and providing maintenance of mucosal homeostasis. However, further studies are required to clarify the role of HIF-1α and IL-33 in the regulation of mucosal barrier integrity and immune response in IBD.

It is claimed that HIF-1α has been linked to myeloid cell function in DSS-induced colitis [107]. Myeloid cells, in particular, are recruited to inflammatory foci and contribute as the body’s first line of defense against infection. Therefore, myeloid hypoxia-induced HIF-1α causes IL-17 secretion to elevate, which, in turn, causes severe colon inflammation in mice with high levels of pro-inflammatory cytokines. Mice with knockout of myeloid HIF-1α (Lyz2-Cre/HIF-1α+f/+f), on the other hand, exhibited moderate colitis and had less invaded immune cells. This is possible because myeloid cells (particularly macrophages) missing HIF-1α have lower motility and invasiveness, which eventually results in a reduction in Th17 level and a decrease in the expression of TNF-α and IFN-γ. These results illustrate the antagonistic role of HIF-1α in immune cells in colon inflammation and suggest that these myeloid cells may play an important role in IBD management. However, another study has already shown that HIF-1α knockout in mice DCs (CD11cCre/HIF-1α+f/+f) results in severe intestinal inflammation with increased levels of pro-inflammatory cytokines and increased production of mucin [48]. In DCs, HIF-1α is required to induce enough Tregs to control bowel inflammation. Meanwhile, Tregs is known to express FoxP3, whose level, in turn, is regulated by HIF-1α [58]. FoxP3 transcription factor expression by Treg cells decreased as a result of the HIF-1α knockout in dendritic cells. Transcriptional response to hypoxia is mainly mediated by epigenetic and post-translational alterations of histone to facilitate transcription of HIF-dependent genes [108]. These contradictory findings, in our opinion, depend on the context of a particular inflammatory lesion and are probably explained by a specific immune response. These outcomes were also attained using a mouse colitis model. To be clear, extensive molecular genetic investigations, including in a large sample of patients with IBD, are required. 

It has been suggested that enhanced expression and activation of the potassium channel K2P5.1 (TASK-2, also known as KCNK5) in CD4+ T-cells in the spleen of mice with DSS-induced colitis could be mediated by inflammation-associated hypoxia [109]. It has been established that hypoxia in inflamed colon tissues causes HIF-1α activation in mouse spleen CD4+ T-cells and encourages the transformation of CD4+ T-cells into Tregs cells, and produce the cytokine IL-10, which is crucial for IBD protection. In the spleen CD4+ T-T cells, it is known that the K2P5.1 channel is the HIF-1α target gene. These finding suggest that K2P5.1 is crucial for the differentiation of Tregs and the induction of IL-10. Further research is needed to elucidate the pathophysiological role of K2P5.1 channels in Tregs, as well as to understand the molecular mechanisms of HIF-1α-mediated K2P5.1. 

**Figure 1:**
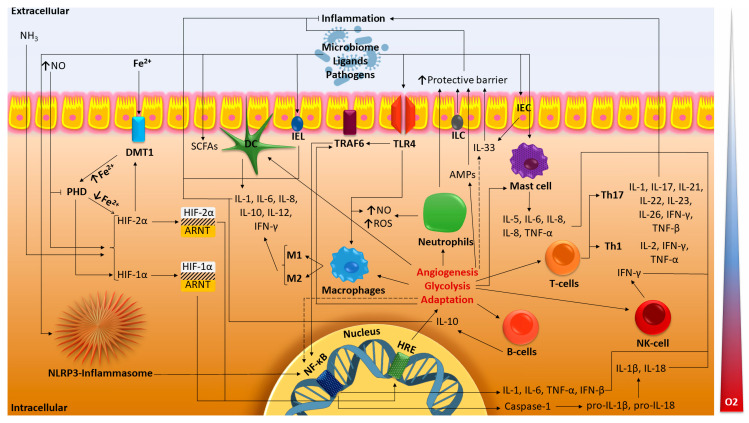
Major molecular pathways of HIF in IBD. The dotted lines indicate the suggested paths. HIF, hypoxia-inducible factor; ARNT, aryl hydrocarbon receptor nuclear translocator; HRE, hypoxia response elements; PHD, prolyl hydroxylase domain; DMT1, divalent metal transporter-1; ROS, reactive oxygen species; NO, nitric oxide; NH_3_, ammonia; IEC, intestinal epithelial cell; IEL, intestinal intraepithelial lymphocyte; ILC, innate lymphoid cell; DC dendritic cell; Th1, Th17, T-helper cells; NK-cell, natural killer T-cell; IL, interleukin; TNF, tumor necrosis factor; IFN, interferon; TLR4, Toll-like receptor 4; TRAF6, TNF receptor-associated factor 6; AMPs, antimicrobial peptides; SCFAs, short-chain fatty acids; NF-κB, nuclear factor κB (original scheme).

HIF-1α may perform a variety of functions across many cell types, and research findings also suggest that HIF-1α may have impacts on intestinal inflammation (Figure 1). It is also highlighted that HIF-1α has the potential to treat intestinal disorders by targeting signaling pathways associated with hypoxia.

#### 3.3.2. Molecular Interactions of HIF-2α in IBD

A number of studies confirm the pro-inflammatory role of HIF-2α in the gut in IBD [9,101,110]. It has been shown that HIF-2α activates neutrophilic inflammation and enhances neutrophil recruitment, contributing to the formation of an inflammatory microenvironment of the colon [110]. In addition, HIF-2-deficient inflammatory neutrophils in mice displayed elevated apoptosis, which caused a decrease of neutrophil-driven inflammation and tissue damage [111]. Accordingly, these findings support a selective role for HIF-2α in maintaining neutrophilic inflammation and provide a platform for considering HIF-2α inhibition as a therapeutic target in chronic inflammatory diseases.

Another study reports that overexpression of the HIF-2 PAS1 endothelial domain protein (EPAS1) led to spontaneous colitis or enhanced susceptibility to DSS-induced colitis in IEC-specific mice with Hif-2a knockouts [112]. Additionally, it was shown that the production of TNF-α in the intestinal epithelium is positively regulated by EPAS1, and that inhibiting TNF-α lessens the inflammation caused by hypoxia in the gut. 

Caveolin-1 (CAV1), a multifunctional protein necessary for occludin metabolism that preserves the tight junction integrity of IECs, has been shown to express itself more when HIF-2α is overexpressed in IECs [113]. Additionally, enhanced endocytosis and intestinal permeability are caused by increased Cav1 expression. HIF2α-mediated signaling increases Cav1 expression, promotes disruption of IEC tight junctions, and increases barrier permeability. The possible new regulatory mechanism for triggering inflammation in hypoxia foci in IBD may be associated with CAV1. However, while this study was carried out in vivo on the HCT116 cell line, it is impossible to extrapolate the findings on a living organism. It is necessary to confirm this mechanism in an animal colitis model. 

It is noted that HIF-2α knockout in macrophages promotes increased expression of IL-6, which is important for the subsequent development of inflammation in the colon [101]. HIF-2 knockout also triggered the production of the pro-inflammatory cytokines IL-23, IL-17, and IFN-γ, as well as a significant increase in neutrophil recruitment carried on by elevated levels of CXCL1 and T-cells. All of these factors combined together may contribute to the progression of colitis. Concurrent induction of IL-10, transforming growth factor beta (TGF-β), and Tregs, however, can slow this process down. Notably, HIF-1a knockout in the same study leads to improvement of acute DSS-colitis. These results demonstrate the different and overlapping functions and immune responses of HIF-1α and HIF-2α in inflammatory bowel disease. Thus, it is predicted that, in the instance of chronic colitis, uncontrolled activation of HIF-2 combined with decreased expression of HIF-1 might result in chronic inflammation and damage.

However, we believe that the available research is insufficient to develop a comprehensive understanding of the processes that result in the specific expression or activation of HIF-2 in the gut in IBD.

#### 3.3.3. Molecular Interactions of PHD Isoforms in IBD

According to reports, PHD1 is activated in colon biopsies from patients with IBD, and its elevated levels are correlated with apoptosis in IBD, especially in UC, as well as with pro-inflammatory markers such as IL-8 and TNF-α [114]. It remains unclear whether pro-inflammatory cytokines influence PHD1 expression or vice versa. It is obvious that the expression of the PHD1 protein as such is not directly connected to its enzymatic activity. However, it is suggested that PHD1 may be a therapeutic target in UC and, to a lesser extent, in colonic CD. These results are supported by other studies where mice deficient in PHD1 but not deficient in PHD2 or PHD3 were protected from DSS-induced colitis by reducing epithelial cell apoptosis and maintaining barrier function [115]. It should be mentioned that DSS-colitis symptoms were less significant when the Phd1 gene was knocked out [116]. The pro-inflammatory signature of endothelial cells was partially reduced in the supernatant from LPS-stimulated Phd1-deficient macrophages, and the production of the pro-inflammatory cytokine IL-1 in response to LPS was decreased in Phd1-deficient DCs. It is well recognized that, in active forms of IBD, the level of IL-1β increases and leads to violation of the epithelial barrier integrity. Therefore, by lowering IL-1β expression, Phd1-deficient macrophages and DCs may work synergistically to maintain barrier function during colitis. Therefore, indeed, inhibition of PHD1 or knockout of the Phd1 gene may represent a potential therapeutic approach for IBD.

The impact of various PHDs on IBD is reviewed. For instance, PHD3 suppression may be linked to UC development and compromised barrier function, as evidenced by the finding that PHD3 levels decrease with increasing disease severity in biopsies from UC patients [117]. PHD3 is required for the synthesis of the occludin protein, which maintains the tight junction integrity of the IECs. It should be highlighted that buildup of HIF-1α and HIF-2α does not arise from a single PHD knockout [118]. This means that since PHD3 has targets other than HIF-1α and HIF-2α, the defect in gut barrier function caused by the loss of PHD3 is not the result of the HIF pathway, but instead is the result of other as yet unidentified mechanisms. 

It has also been discovered that TLR4 signaling increases PHD3 expression in DCs in response to LPS stimulation [119]. These data raise questions about PHD3’s potential therapeutic value in inflammation, while demonstrating a cell-specific competitive advantage of PHD3 expression in antigen-presenting cells. As noted earlier, TLR4 plays an important role in the pathogenesis of IBD. According to reports, TLR4 can affect whether IBD becomes better or gets worse, and also, taking into consideration the fact that LPS promotes the expression of HIF-1α and is a TLR4 ligand, we can assume the possible existence of HIF-1α/TLR4 cross-signaling pathways [12]. This approach will allow us to look at the potential involvement and function of HIF-1α/TLR4 for LPS-induced inflammation in IBD.

It is clear that diverse PHD isoforms exhibit multiple effects on intestinal inflammation. Particularly, PHD1 inhibition or HIF-1α activity increase are thought to be effective treatments for IBD; conversely, PHD3 activity decrease or HIF-2α stability enhancement may have detrimental effects on IBD. We therefore believe that, depending on the context of a certain inflammatory lesion, a clear understanding of the molecular interaction’s mechanisms of various PHD isoforms in the HIF-1α and HIF-2α signaling pathways is required for the beneficial use of PHD for IBD therapy.

### 3.4. Possible IBD Therapy by Targeting HIF

Concerning the development of novel therapeutic strategies for the treatment of intestinal inflammation, pharmacological effects on hypoxia signaling pathways are a research area of interest. In particular, stabilization of HIF-1α has been reported to possess a positive effect on mucosal health [102]. Inflammation-related stabilization of HIF-1α1 results in the induction of a number of protective molecules, as was already mentioned. Therefore, pharmaceutical HIF-1α activation and stabilization may be beneficial for the treatment of IBD. However, the pleiotropic effects brought on by HIF-1α’s direct regulation of transcription raises the possibility of incidental or unexpected side effects. HIF-1α activation, nevertheless, may be protective in IBD according to findings. Therefore, PHDi-based molecular therapies for IBD are being researched on the basis of inhibitors of prolyl hydroxylase (PHI, PHDi) of the co-substrate 2-oxoglutarate as controlling the expression of HIF-1α [120]. While 2-oxoglutarate analogues’ pharmacological inhibition of PHDi is adequate to stabilize HIF-1α, it is not specific to distinct PHD isoforms. For example, dimethyloxalylglycine (DMOG), a cell-permeable competitive inhibitor of HIF-PH, is a panhydroxylase inhibitor that can impede the activity of PHDi and asparaginyl hydroxylase (FIH) [121]. DMOG provides a barrier protective effect and slows down disease progression. It has also been shown that the combination action of DMOG and the anti-inflammatory medication cyclosporine may produce a putatively improved protective effect by concurrently acting on inflammation and barrier failure. As a result, the combination of the two drugs generated a stronger adaptive protective effect than either one of them would have on its own. It is hypothesized that the maintenance of the barrier function and, in part, the control of the protein Zonula occludens-1, also known as TJP1, were responsible for DMOG’s capacity to improve the anti-inflammatory effects of cyclosporine. It is important to note that research on targeted DMOG administration has demonstrated improved medication effectiveness with less systemic exposure and decreased risk of unfavorable outcomes in a DSS model of colitis [122].

PHDi inhibition with the AKB-4924 (selective stabilizer of HIF-1α, also known as GB004) has demonstrated an enhancement of the innate immune response as well as a central role for the epithelium in the anti-inflammatory protection. These data were obtained in mice with 2,4,6-trinitrobenzenesulfonic acid (TNBS)-induced colitis [123]. AKB-4924 treatment improved epithelial barrier function and decreased serum levels of the endotoxin LPS in TNBS-colitis. It should be noted that AKB-4924 also decreased serum levels of IL-1, IL-6, and TNF-, but increased IL-10 levels. In epithelium-specific mice with HIF-1α deficiency, the therapy did not prevent colitis, indicating that epithelial HIF-1α is a tissue target for AKB-4924-mediated protection. 

The inhibition of cullin’s neddylation is shown to be a potential alternative method for stabilizing HIF-1α [124]. Neddylation, also known as modification with neural precursor cell expressed, developmentally down-regulated 8 (NEDD8), causes the NEDD8 residue to bind covalently to the conserved lysine residue of cullin, which helps to stabilize the protein. The stability and destruction of HIF proteins via Cullin-2 depends on NEDD8 targets from the cullin family. The NEDD8-activating enzyme (NAE), which is inhibited by the small-molecule inhibitor MLN4924, a structural analog of adenosine monophosphate, prevents the neddylation of cullin proteins, which is necessary for the degradation of HIF-1α. In IECs, it was demonstrated that pharmacological influence on MLN4924 activity stabilized HIF-1α, activated the HIF promoter, and activatedinduced HIF target genes. Consequently, MLN4924 stabilizes HIF in a way that is analogous to PHDi. 

It is reported that treating another HIF inhibitor, TRC160334, which activates HIF transcription in a dose-dependent manner, decreases colitis in animals [125]. The cytoprotective Heat Shock Protein 70 (HSP70) was significantly produced in the colon after treatment with TRC160334, along with a decrease in the expression of TNF-α and INF-γ mRNA and an increase in the expression of the anti-inflammatory cytokine IL-10. These results demonstrate the therapeutic potential of TRC160334 for the treatment of IBD.

Another potential target for IBD therapy is HIF-2α [126]. Small inhibitor molecules can be inserted into the interior hydrophobic cavity of the HIF-2α PAS-B domain, which may be helpful for ligand-mediated allosteric regulation in inflammatory disorders [127]. However, it is essential to keep in mind that these inhibitors can also lead to HIF-2α mediated inflammation, which is highly harmful in IBDs. Therefore, more research is necessary to determine the efficiency of potential therapy of this kind.

Histone deacetylase (HDAC) inhibitors are well known for being significant inflammatory regulators. In mice with DSS-induced colitis, the HDAC inhibitor suberoylanilide hydroxamic acid (SAHA) has been shown to lessen disease severity by preventing the release of pro-inflammatory cytokines and chemokines [128]. In particular, SAHA therapy reduced dramatically the production of the cytokines IL-6, TNF-, and CCL2, as well as the quantity of migratory inflammatory cells. However, more investigation is required to completely understand the molecular pathways SAHA’s influence on the pathogenesis of IBD.

Moreover, data were represented that demonstrate how hypoxia can lessen intestinal inflammation by inhibiting the nucleotide-binding domain of oligomerization NLRP3, which is the rapamycin target pathway (mTOR), and autophagy activation [129]. As a result, the role of NLRP3 in the regulation of hypoxia-mediated anti-inflammatory outcomes throughout NLRP3 clearance and subsequent activation of autophagy and reduction of inflammatory processes in IECs has been confirmed. At the same time, NLRP3 degradation caused by hypoxia precludes stimulatory effects on mTOR signaling and also restricts NLRP3 inflammation-dependent responses. Inflammasomes reduce the clinical symptoms of colitis in response to inflammation and then prevent additional injury. This finding potentially benefits in the development of future, potent therapeutic therapies for the treatment of IBD. However, according to other studies, NLRP3 could indeed make colon damage from IBD worse [130]. It was discovered, in particular, that a loss-of-function mutation in the CARD8 protein, which normally suppresses the NLRP3 inflammasome, causes it to activate and contributes in CD progression [131]. Studies on various research subjects, including IBD patients and experimentally model colitis in animals, can be used to verify these discrepancies. Further research in IBD patients and healthy individuals is required to understand the precise function of the NLRP3 inflammasome in IBD as well as in the non-inflammatory intestinal mucosa. It is crucial to examine the precise mechanisms of NLRP3-mediated inflammation in a sample of IBD patients because a model of colitis cannot accurately imitate all clinical manifestations and mechanisms of IBD in humans. 

It might be said that the IBD therapy that is currently being investigated by acting on HIF is promising. However, the inducing and cell-specific effects of HIF-1α signaling, as well as how these effects are influenced by the tissue microenvironment, must be taken into account when developing therapies that target HIF-1α. It is essential to have a comprehensive understanding of the mechanisms underlying these effects, in addition to how the tissue context and interactions with partner proteins affect them. Additionally, the possibility of negative side effects in IBD is increased by the systemic treatment of hydroxylase inhibitors. Therefore, it is critical to create targeted drug delivery strategies that enable local drug administration to the intestine’s afflicted sections with little risk of systemic exposure.

## 4. Further Perspectives and Conclusive Remarks

It is obvious that HIF is crucial for intestinal hypoxia. The pathways involved in the activation of HIF-1 and HIF-2 are intricate and multifactorial, and each has a remarkable effect on how inflammatory diseases develop and change over time. Most research has indicated that HIF-1 has therapeutic potential and can be used to target signaling pathways associated with intestinal disorders with hypoxia. According to some experts, HIF-2 plays a more pro-inflammatory role. However, because it is still unclear exactly how the HIF-1 and HIF-2 signaling pathways are coordinated in inflammatory conditions, and the potential effects of cross-signaling immune cell pathways have not yet been clearly established, research results do not conclusively establish the role of HIF-1 and HIF-2 in intestinal inflammation. To make sense of the evidence already available, resolve any inconsistencies that have emerged, and confirm or deny the suggested conjectures, more research studies are necessary. 

However, research examining the effect of HIF activation by pharmacological intervention on hypoxia signaling pathways has produced encouraging findings that bring us closer to the creation of novel therapeutics for the treatment of intestinal inflammation. Looking to exploit the potential for protection of oxygen-sensitive pathways requires the development of additional treatment strategies. In the upcoming years, it is hoped that we will reach a step closer to comprehending the molecular mechanisms underpinning HIF signaling in the gut in IBD.

## Data Availability

No new data were created or analyzed in this study. Data sharing is not applicable to this article.

## Abbreviations

IBD	Inflammatory bowel disease
CD	Crohn’s disease
UC	Ulcerative colitis
GI	Gastrointestinal tract
pO_2_	Partial pressure
HIF	Hypoxia-inducible factor
ARNT	Aryl hydrocarbon receptor nuclear translocator
HRE	Hypoxia response elements
PHD	Prolyl hydroxylase domain
FIH	Factor inhibiting HIF
IDH	Isocitrate dehydrogenase
FPN	Basolateral iron transporter ferroportin
ROS	Reactive oxygen species
IECs	Intestinal epithelial cells
IELs	Intestinal intraepithelial lymphocytes
ILCs	Innate lymphoid cells
DCs	Dendritic cells
Th cells	T-helper cells
Tregs	Regulatory T-cells
NK-cells	Natural killer T-cells
IL	Interleukin
TNF	Tumor necrosis factor
TGF-β	Transforming growth factor beta
IFN	Interferon
TLRs	Toll-like receptors
AMPs	Antimicrobial peptides
SCFAs	Short chain fatty acids
DSS	Dextran sulfate sodium
PRRs	Pattern recognition receptors
PAMPs	Pathogen-associated molecular patterns
LPS	Lipopolysaccharide
FoxP3	Tregs forkhead box P3
NF-κB	Nuclear factor κB
NADPH 1	Nicotinamide adenine dinucleotide phosphate oxidase complex Nox1
DMOG	Dimethyloxalylglycine
PHDi	Prolyl hydroxylase inhibitors
SAHA	Suberoylanilide hydroxamic acid

## References

[1] Konjar Š., Pavšič M., Veldhoen M. (2021). Regulation of Oxygen Homeostasis at the Intestinal Epithelial Barrier Site. Int. J. Mol. Sci..

[2] Ortiz-Prado E., Dunn J.F., Vasconez J., Castillo D., Viscor G. (2019). Partial Pressure of Oxygen in the Human Body: A General Review. Am. J. Blood Res..

[3] Reyman M., van Houten M.A., van Baarle D., Bosch A.A.T.M., Man W.H., Chu M.L.J.N., Arp K., Watson R.L., Sanders E.A.M., Fuentes S. (2019). Impact of delivery mode-associated gut microbiota dynamics on health in the first year of life. Nat. Commun..

[4] Lee J.Y., Tsolis R.M., Bäumler A.J. (2022). The microbiome and gut homeostasis. Science.

[5] Sommer F., Anderson J.M., Bharti R., Raes J., Rosenstiel P. (2017). The resilience of the intestinal microbiota influences health and disease. Nat. Rev. Microbiol..

[6] Lerner A., Shoenfeld Y., Matthias T. (2017). Adverse effects of gluten ingestion and advantages of gluten withdrawal in nonceliac autoimmune disease. Nutr. Rev..

[7] Rivera-Chávez F., Lopez C.A., Bäumler A.J. (2017). Oxygen as a Driver of Gut Dysbiosis. Free. Radic. Biol. Med..

[8] Yang C., Zhong Z.-F., Wang S.-P., Vong C.-T., Yu B., Wang Y.-T. (2021). HIF-1: Structure, Biology and Natural Modulators. Chin. J. Nat. Med..

[9] Ramakrishnan S.K., Shah Y.M. (2016). Role of Intestinal HIF-2α in Health and Disease. Annu. Rev. Physiol..

[10] Sun L., Li T., Tang H., Yu K., Ma Y., Yu M., Qiu Y., Xu P., Xiao W., Yang H. (2019). Intestinal Epithelial Cells-Derived Hypoxia-Inducible Factor-1α Is Essential for the Homeostasis of Intestinal Intraepithelial Lymphocytes. Front. Immunol..

[11] Dengler V.L., Galbraith M., Espinosa J.M. (2014). Transcriptional Regulation by Hypoxia Inducible Factors. Crit. Rev. Biochem. Mol. Biol..

[12] Watts E.R., Walmsley S.R. (2019). Inflammation and Hypoxia: HIF and PHD Isoform Selectivity. Trends Mol. Med..

[13] Singhal R., Shah Y.M. (2020). Oxygen Battle in the Gut: Hypoxia and Hypoxia-Inducible Factors in Metabolic and Inflammatory Responses in the Intestine. J. Biol. Chem..

[14] Heir P., Ohh M., Ursini-Siegel J., Beauchemin N. (2016). Hydroxylation-Dependent Interaction of Substrates to the Von Hippel-Lindau Tumor Suppressor Protein (VHL). The Tumor Microenvironment: Methods and Protocols.

[15] Volkova Y.L., Pickel C., Jucht A.E., Wenger R.H., Scholz C.C. (2022). The Asparagine Hydroxylase FIH: A Unique Oxygen Sensor. Antioxid. Redox Signal..

[16] Du X., Hu H. (2021). The Roles of 2-Hydroxyglutarate. Front. Cell Dev. Biol..

[17] Tannahill G.M., Curtis A.M., Adamik J., Palsson-McDermott E.M., McGettrick A.F., Goel G., Frezza C., Bernard N.J., Kelly B., Foley N.H. (2013). Succinate Is an Inflammatory Signal That Induces IL-1β through HIF-1α. Nature.

[18] Mastrogiannaki M., Matak P., Peyssonnaux C. (2013). The Gut in Iron Homeostasis: Role of HIF-2 under Normal and Pathological Conditions. Blood.

[19] Schwartz A.J., Das N.K., Ramakrishnan S.K., Jain C., Jurkovic M.T., Wu J., Nemeth E., Lakhal-Littleton S., Colacino J.A., Shah Y.M. (2019). Hepatic Hepcidin/Intestinal HIF-2α Axis Maintains Iron Absorption during Iron Deficiency and Overload. J. Clin. Investig..

[20] Martínez-Reyes I., Diebold L.P., Kong H., Schieber M., Huang H., Hensley C.T., Mehta M.M., Wang T., Santos J.H., Woychik R. (2016). TCA Cycle and Mitochondrial Membrane Potential Are Necessary for Diverse Biological Functions. Mol. Cell.

[21] Gonzalez F.J., Xie C., Jiang C. (2019). The Role of Hypoxia-Inducible Factors in Metabolic Diseases. Nat. Rev. Endocrinol..

[22] Xie C., Yagai T., Luo Y., Liang X., Chen T., Wang Q., Sun D., Zhao J., Ramakrishnan S.K., Sun L. (2017). Activation of Intestinal Hypoxia-Inducible Factor 2α during Obesity Contributes to Hepatic Steatosis. Nat. Med..

[23] Kalantar-Zadeh K., Berean K.J., Burgell R.E., Muir J.G., Gibson P.R. (2019). Intestinal Gases: Influence on Gut Disorders and the Role of Dietary Manipulations. Nat. Rev. Gastroenterol. Hepatol..

[24] Zhao Y., Wang X., Noviana M., Hou M. (2018). Nitric oxide in red blood cell adaptation to hypoxia. Acta Biochim. Biophys. Sin..

[25] Lercher A., Baazim H., Bergthaler A. (2020). Systemic Immunometabolism: Challenges and Opportunities. Immunity.

[26] Colgan S.P., Furuta G.T., Taylor C.T. (2020). Hypoxia and Innate Immunity: Keeping Up with the HIFsters. Annu. Rev. Immunol..

[27] Taylor C.T., Doherty G., Fallon P.G., Cummins E.P. (2016). Hypoxia-Dependent Regulation of Inflammatory Pathways in Immune Cells. J. Clin. Investig..

[28] Corcoran S.E., O’Neill L.A.J. (2016). HIF1α and Metabolic Reprogramming in Inflammation. J. Clin. Investig..

[29] Jasper H. (2020). Intestinal Stem Cell Aging: Origins and Interventions. Annu. Rev. Physiol..

[30] Steiner C.A., Cartwright I.M., Taylor C.T., Colgan S.P. (2022). Hypoxia-Inducible Factor as a Bridge between Healthy Barrier Function, Wound Healing, and Fibrosis. Am. J. Physiol. Cell Physiol..

[31] Olivares-Villagómez D., Van Kaer L. (2018). Intestinal Intraepithelial Lymphocytes: Sentinels of the Mucosal Barrier. Trends Immunol..

[32] Allaire J.M., Crowley S.M., Law H.T., Chang S.-Y., Ko H.-J., Vallance B.A. (2018). The Intestinal Epithelium: Central Coordinator of Mucosal Immunity. Trends Immunol..

[33] Birchenough G.M.H., Johansson M.E.V., Gustafsson J.K., Bergström J.H., Hansson G.C. (2015). New Developments in Goblet Cell Mucus Secretion and Function. Mucosal Immunol..

[34] Bin Hafeez A., Jiang X., Bergen P.J., Zhu Y. (2021). Antimicrobial Peptides: An Update on Classifications and Databases. Int. J. Mol. Sci..

[35] Louis N.A., Hamilton K.E., Canny G., Shekels L.L., Ho S.B., Colgan S.P. (2006). Selective Induction of Mucin-3 by Hypoxia in Intestinal Epithelia. J. Cell. Biochem..

[36] Krzywinska E., Stockmann C. (2018). Hypoxia, Metabolism and Immune Cell Function. Biomedicines.

[37] Saeedi B.J., Kao D.J., Kitzenberg D.A., Dobrinskikh E., Schwisow K.D., Masterson J.C., Kendrick A.A., Kelly C.J., Bayless A.J., Kominsky D.J. (2015). HIF-Dependent Regulation of Claudin-1 Is Central to Intestinal Epithelial Tight Junction Integrity. Mol. Biol. Cell.

[38] Benjamin J.L., Sumpter R., Levine B., Hooper L.V. (2013). Intestinal Epithelial Autophagy Is Essential for Host Defense against Invasive Bacteria. Cell Host Microbe.

[39] Fellows R., Denizot J., Stellato C., Cuomo A., Jain P., Stoyanova E., Balázsi S., Hajnády Z., Liebert A., Kazakevych J. (2018). Microbiota Derived Short Chain Fatty Acids Promote Histone Crotonylation in the Colon through Histone Deacetylases. Nat. Commun..

[40] Zhou C., Li L., Li T., Sun L., Yin J., Guan H., Wang L., Zhu H., Xu P., Fan X. (2020). SCFAs Induce Autophagy in Intestinal Epithelial Cells and Relieve Colitis by Stabilizing HIF-1α. J. Mol. Med..

[41] Fitzgerald K.A., Kagan J.C. (2020). Toll-like Receptors and the Control of Immunity. Cell.

[42] McKernan D.P., Kumar V. (2022). Toll-Like Receptors as Drug Targets in the Intestinal Epithelium. Toll-Like Receptors in Health and Disease.

[43] Zhang J., Han C., Dai H., Hou J., Dong Y., Cui X., Xu L., Zhang M., Xia Q. (2016). Hypoxia-Inducible Factor-2α Limits Natural Killer T Cell Cytotoxicity in Renal Ischemia/Reperfusion Injury. J. Am. Soc. Nephrol..

[44] Mills E.L., O’Neill L.A. (2016). Reprogramming Mitochondrial Metabolism in Macrophages as an Anti-Inflammatory Signal. Eur. J. Immunol..

[45] Domblides C., Lartigue L., Faustin B. (2018). Metabolic Stress in the Immune Function of T Cells, Macrophages and Dendritic Cells. Cells.

[46] Palsson-McDermott E.M., Curtis A.M., Goel G., Lauterbach M.A.R., Sheedy F.J., Gleeson L.E., van den Bosch M.W.M., Quinn S.R., Domingo-Fernandez R., Johnston D.G.W. (2015). Pyruvate Kinase M2 Regulates Hif-1α Activity and IL-1β Induction and Is a Critical Determinant of the Warburg Effect in LPS-Activated Macrophages. Cell Metab..

[47] Bošnjak B., Do K.T.H., Förster R., Hammerschmidt S.I. (2022). Imaging Dendritic Cell Functions. Immunol. Rev..

[48] Flück K., Breves G., Fandrey J., Winning S. (2016). Hypoxia-Inducible Factor 1 in Dendritic Cells Is Crucial for the Activation of Protective Regulatory T Cells in Murine Colitis. Mucosal Immunol..

[49] Wobben R., Hüsecken Y., Lodewick C., Gibbert K., Fandrey J., Winning S. (2013). Role of Hypoxia Inducible Factor-1α for Interferon Synthesis in Mouse Dendritic Cells. Biol. Chem..

[50] Walmsley S.R., Chilvers E.R., Thompson A.A., Vaughan K., Marriott H.M., Parker L.C., Shaw G., Parmar S., Schneider M., Sabroe I. (2011). Prolyl Hydroxylase 3 (PHD3) Is Essential for Hypoxic Regulation of Neutrophilic Inflammation in Humans and Mice. J. Clin. Investig..

[51] Campbell E.L., Bruyninckx W.J., Kelly C.J., Glover L.E., McNamee E.N., Bowers B.E., Bayless A.J., Scully M., Saeedi B.J., Golden-Mason L. (2014). Transmigrating Neutrophils Shape the Mucosal Microenvironment through Localized Oxygen Depletion to Influence Resolution of Inflammation. Immunity.

[52] Loftus R.M., Finlay D.K. (2016). Immunometabolism: Cellular Metabolism Turns Immune Regulator. J. Biol. Chem..

[53] Bagadia P., Huang X., Liu T.-T., Murphy K.M. (2019). Shared Transcriptional Control of Innate Lymphoid Cell and Dendritic Cell Development. Annu. Rev. Cell Dev. Biol..

[54] Li Q., Li D., Zhang X., Wan Q., Zhang W., Zheng M., Zou L., Elly C., Lee J.H., Liu Y.-C. (2018). E3 Ligase VHL Promotes Group 2 Innate Lymphoid Cell Maturation and Function via Glycolysis Inhibition and Induction of Interleukin-33 Receptor. Immunity.

[55] Burrows N., Maxwell P.H. (2017). Hypoxia and B Cells. Exp. Cell Res..

[56] Barbi J., Pardoll D., Pan F. (2013). Metabolic Control of the Treg/Th17 Axis. Immunol. Rev..

[57] Shehade H., Acolty V., Moser M., Oldenhove G. (2015). Cutting Edge: Hypoxia-Inducible Factor 1 Negatively Regulates Th1 Function. J. Immunol..

[58] Colamatteo A., Carbone F., Bruzzaniti S., Galgani M., Fusco C., Maniscalco G.T., Di Rella F., de Candia P., De Rosa V. (2019). Molecular Mechanisms Controlling Foxp3 Expression in Health and Autoimmunity: From Epigenetic to Post-Translational Regulation. Front. Immunol..

[59] Lee J.H., Elly C., Park Y., Liu Y.-C. (2015). E3 Ubiquitin Ligase VHL Regulates Hypoxia-Inducible Factor-1α to Maintain Regulatory T Cell Stability and Suppressive Capacity. Immunity.

[60] Shi L.Z., Wang R., Huang G., Vogel P., Neale G., Green D.R., Chi H. (2011). HIF1alpha-Dependent Glycolytic Pathway Orchestrates a Metabolic Checkpoint for the Differentiation of TH17 and Treg Cells. J. Exp. Med..

[61] Palazon A., Tyrakis P.A., Macias D., Veliça P., Rundqvist H., Fitzpatrick S., Vojnovic N., Phan A.T., Loman N., Hedenfalk I. (2017). An HIF-1α/VEGF-A Axis in Cytotoxic T Cells Regulates Tumor Progression. Cancer Cell.

[62] Shin D.H., Lin H., Zheng H., Kim K.S., Kim J.Y., Chun Y.S., Park J.W., Nam J.H., Kim W.K., Zhang Y.H. (2014). HIF-1α–Mediated Upregulation of TASK-2 K^+^ Channels Augments Ca^2+^ Signaling in Mouse B Cells under Hypoxia. J. Immunol..

[63] Cho S.H., Raybuck A.L., Stengel K., Wei M., Beck T.C., Volanakis E., Thomas J.W., Hiebert S., Haase V.H., Boothby M.R. (2016). Germinal Centre Hypoxia and Regulation of Antibody Qualities by a Hypoxia Response System. Nature.

[64] Meng X., Grötsch B., Luo Y., Knaup K.X., Wiesener M.S., Chen X.-X., Jantsch J., Fillatreau S., Schett G., Bozec A. (2018). Hypoxia-Inducible Factor-1α Is a Critical Transcription Factor for IL-10-Producing B Cells in Autoimmune Disease. Nat. Commun..

[65] Hams E., Saunders S.P., Cummins E.P., O’Connor A., Tambuwala M.T., Gallagher W.M., Byrne A., Campos-Torres A., Moynagh P.M., Jobin C. (2011). The Hydroxylase Inhibitor Dimethyloxallyl Glycine Attenuates Endotoxic Shock via Alternative Activation of Macrophages and IL-10 Production by B1 Cells. Shock.

[66] Korbecki J., Simińska D., Gąssowska-Dobrowolska M., Listos J., Gutowska I., Chlubek D., Baranowska-Bosiacka I. (2021). Chronic and Cycling Hypoxia: Drivers of Cancer Chronic Inflammation through HIF-1 and NF-ΚB Activation: A Review of the Molecular Mechanisms. Int. J. Mol. Sci..

[67] Scholz C.C., Cavadas M.A.S., Tambuwala M.M., Hams E., Rodríguez J., von Kriegsheim A., Cotter P., Bruning U., Fallon P.G., Cheong A. (2013). Regulation of IL-1β-Induced NF-ΚB by Hydroxylases Links Key Hypoxic and Inflammatory Signaling Pathways. Proc. Natl. Acad. Sci. USA.

[68] D’Ignazio L., Shakir D., Batie M., Muller H.A., Rocha S. (2020). HIF-1β Positively Regulates NF-ΚB Activity via Direct Control of TRAF6. Int. J. Mol. Sci..

[69] Dong S., Liang S., Cheng Z., Zhang X., Luo L., Li L., Zhang W., Li S., Xu Q., Zhong M. (2022). ROS/PI3K/Akt and Wnt/β-Catenin Signalings Activate HIF-1α-Induced Metabolic Reprogramming to Impart 5-Fluorouracil Resistance in Colorectal Cancer. J. Exp. Clin. Cancer Res..

[70] Agrawal M., Allin K.H., Petralia F., Colombel J.-F., Jess T. (2022). Multiomics to Elucidate Inflammatory Bowel Disease Risk Factors and Pathways. Nat. Rev. Gastroenterol. Hepatol..

[71] Burke K.E., D’Amato M., Ng S.C., Pardi D.S., Ludvigsson J.F., Khalili H. (2021). Microscopic colitis. Nat. Rev. Dis. Prim..

[72] Kofla-Dłubacz A., Pytrus T., Akutko K., Sputa-Grzegrzółka P., Piotrowska A., Dzięgiel P. (2022). Etiology of IBD-Is It Still a Mystery?. Int. J. Mol. Sci..

[73] Odenwald M.A., Turner J.R. (2017). The Intestinal Epithelial Barrier: A Therapeutic Target?. Nat. Rev. Gastroenterol. Hepatol..

[74] Lee M., Chang E.B. (2021). Inflammatory Bowel Diseases (IBD) and the Microbiome—Searching the Crime Scene for Clues. Gastroenterology.

[75] Binion D.G. (2012). Clostridium Difficile Infection in Patients with Inflammatory Bowel Disease. Gastroenterol. Hepatol..

[76] Wah-Suárez M.I., Vázquez M.A.M., Bosques-Padilla F.J. (2022). Inflammatory Bowel Disease: The Role of Commensal Microbiome in Immune Regulation. Gastroenterol. Hepatol..

[77] Mirsepasi-Lauridsen H.C., Du Z., Struve C., Charbon G., Karczewski J., Krogfelt K.A., Wells J.M. (2016). Secretion of Alpha-Hemolysin by Escherichia coli Disrupts Tight Junctions in Ulcerative Colitis Patients. Clin. Transl. Gastroenterol..

[78] Wagner J., Skinner N.A., Catto-Smith A.G., Cameron D.J., Michalski W.P., Visvanathan K., Kirkwood C.D. (2013). TLR4, IL10RA, and NOD2 mutation in paediatric Crohn’s disease patients: An association with Mycobacterium avium subspecies paratuberculosis and TLR4 and IL10RA expression. Med. Microbiol. Immunol..

[79] Gevers D., Kugathasan S., Denson L.A., Vázquez-Baeza Y., Van Treuren W., Ren B., Xavier R.J. (2014). The treatment-naive microbiome in new-onset Crohn’s disease. Cell Host Microbe.

[80] Mirsepasi-Lauridsen H.C., Vrankx K., Engberg J., Friis-Møller A., Brynskov J., Nordgaard-Lassen I., Krogfelt K.A. (2018). Disease-Specific Enteric Microbiome Dysbiosis in Inflammatory Bowel Disease. Front. Med..

[81] Turner D., Bishai J., Reshef L., Abitbol G., Focht G., Marcus D., Ledder O., Lev-Tzion R., Orlanski-Meyer E., Yerushalmi B. (2020). Antibiotic Cocktail for Pediatric Acute Severe Colitis and the Microbiome: The PRASCO Randomized Controlled Trial. Inflamm. Bowel Dis..

[82] Pigneur B., Lepage P., Mondot S., Schmitz J., Goulet O., Doré J., Ruemmele F.M. (2019). Mucosal Healing and Bacterial Composition in Response to Enteral Nutrition Vs Steroid-Based Induction Therapy-A Randomised Prospective Clinical Trial in Children with Crohn’s Disease. J. Crohn’s Colitis.

[83] Milajerdi A., Sadeghi O., Siadat S.D., Keshavarz S.A., Sima A., Vahedi H., Adibi P., Esmaillzadeh A. (2020). A Randomized Controlled Trial Investigating the Effect of a Diet Low in Fermentable Oligosaccharides, Disaccharides, Monosaccharides, and Polyols on the Intestinal Microbiome and Inflammation in Patients with Ulcerative Colitis: Study Protocol for a Randomized Controlled Trial. Trials.

[84] Nissilä E., Korpela K., Lokki A.I., Paakkanen R., Jokiranta S., de Vos W.M., Lokki M.-L., Kolho K.-L., Meri S. (2017). C4B Gene Influences Intestinal Microbiota through Complement Activation in Patients with Paediatric-Onset Inflammatory Bowel Disease. Clin. Exp. Immunol..

[85] Schwerd T., Bryant R.V., Pandey S., Capitani M., Meran L., Cazier J.-B., Jung J., Mondal K., Parkes M., Mathew C.G. (2018). NOX1 Loss-of-Function Genetic Variants in Patients with Inflammatory Bowel Disease. Mucosal Immunol..

[86] Meir M., Flemming S., Burkard N., Bergauer L., Metzger M., Germer C.-T., Schlegel N. (2015). Glial Cell Line-Derived Neurotrophic Factor Promotes Barrier Maturation and Wound Healing in Intestinal Epithelial Cells in Vitro. Am. J. Physiol. Gastrointest. Liver Physiol..

[87] Meir M., Burkard N., Ungewiß H., Diefenbacher M., Flemming S., Kannapin F., Germer C.-T., Schweinlin M., Metzger M., Waschke J. (2019). Neurotrophic Factor GDNF Regulates Intestinal Barrier Function in Inflammatory Bowel Disease. J. Clin. Investig..

[88] Pelaseyed T., Bergström J.H., Gustafsson J.K., Ermund A., Birchenough G.M.H., Schütte A., van der Post S., Svensson F., Rodríguez-Piñeiro A.M., Nyström E.E.L. (2014). The Mucus and Mucins of the Goblet Cells and Enterocytes Provide the First Defense Line of the Gastrointestinal Tract and Interact with the Immune System. Immunol. Rev..

[89] Engevik M.A., Luk B., Chang-Graham A.L., Hall A., Herrmann B., Ruan W., Endres B.T., Shi Z., Garey K.W., Hyser J.M. (2019). Bifidobacterium Dentium Fortifies the Intestinal Mucus Layer via Autophagy and Calcium Signaling Pathways. mBio.

[90] Mukherjee T., Hovingh E.S., Foerster E.G., Abdel-Nour M., Philpott D.J., Girardin S.E. (2019). NOD1 and NOD2 in Inflammation, Immunity and Disease. Arch. Biochem. Biophys..

[91] Strober W., Watanabe T. (2011). NOD2, an Intracellular Innate Immune Sensor Involved in Host Defense and Crohn’s Disease. Mucosal Immunol..

[92] Roda G., Chien Ng S., Kotze P.G., Argollo M., Panaccione R., Spinelli A., Kaser A., Peyrin-Biroulet L., Danese S. (2020). Crohn’s Disease. Nat. Rev. Dis. Prim..

[93] Yan J.-B., Luo M.-M., Chen Z.-Y., He B.-H. (2020). The Function and Role of the Th17/Treg Cell Balance in Inflammatory Bowel Disease. J. Immunol. Res..

[94] Mao L., Kitani A., Strober W., Fuss I.J. (2018). The Role of NLRP3 and IL-1β in the Pathogenesis of Inflammatory Bowel Disease. Front. Immunol..

[95] Dai W., Long L., Wang X., Li S., Xu H. (2022). Phytochemicals Targeting Toll-like Receptors 4 (TLR4) in Inflammatory Bowel Disease. Chin. Med..

[96] Chen Z., Zhang Y., Lin R., Meng X., Zhao W., Shen W., Fan H. (2020). Cronobacter Sakazakii Induces Necrotizing Enterocolitis by Regulating NLRP3 Inflammasome Expression via TLR4. J. Med. Microbiol..

[97] McKernan D.P., Finn D.P. (2014). An ApPEAling New Therapeutic for Ulcerative Colitis?. Gut.

[98] Taylor C.T. (2017). Hypoxia in the Gut. Cell. Mol. Gastroenterol. Hepatol..

[99] Colgan S.P., Campbell E.L., Kominsky D.J. (2016). Hypoxia and Mucosal Inflammation. Annu. Rev. Pathol..

[100] Brown E., Taylor C.T. (2018). Hypoxia-sensitive Pathways in Intestinal Inflammation. J. Physiol..

[101] Kerber E.L., Padberg C., Koll N., Schuetzhold V., Fandrey J., Winning S. (2020). The Importance of Hypoxia-Inducible Factors (HIF-1 and HIF-2) for the Pathophysiology of Inflammatory Bowel Disease. Int. J. Mol. Sci..

[102] Yin J., Ren Y., Yang K., Wang W., Wang T., Xiao W., Yang H. (2022). The Role of Hypoxia-Inducible Factor 1-Alpha in Inflammatory Bowel Disease. Cell Biol. Int..

[103] Pazmandi J., Kalinichenko A., Ardy R.C., Boztug K. (2019). Early-onset Inflammatory Bowel Disease as a Model Disease to Identify Key Regulators of Immune Homeostasis Mechanisms. Immunol. Rev..

[104] Zhu L., Shi T., Zhong C., Wang Y., Chang M., Liu X. (2017). IL-10 and IL-10 Receptor Mutations in Very Early Onset Inflammatory Bowel Disease. Gastroenterol. Res..

[105] Sukumar M., Liu J., Ji Y., Subramanian M., Crompton J.G., Yu Z., Roychoudhuri R., Palmer D.C., Muranski P., Karoly E.D. (2013). Inhibiting Glycolytic Metabolism Enhances CD8+ T Cell Memory and Antitumor Function. J. Clin. Investig..

[106] Sun M., He C., Wu W., Zhou G., Liu F., Cong Y., Liu Z. (2017). Hypoxia Inducible Factor-1α-Induced Interleukin-33 Expression in Intestinal Epithelia Contributes to Mucosal Homeostasis in Inflammatory Bowel Disease. Clin. Exp. Immunol..

[107] Bäcker V., Cheung F.-Y., Siveke J.T., Fandrey J., Winning S. (2017). Knockdown of Myeloid Cell Hypoxia-Inducible Factor-1α Ameliorates the Acute Pathology in DSS-Induced Colitis. PLoS ONE.

[108] Choudhry H., Harris A.L. (2018). Advances in Hypoxia-Inducible Factor Biology. Cell Metab..

[109] Endo K., Kito H., Tanaka R., Kajikuri J., Tanaka S., Elboray E.E., Suzuki T., Ohya S. (2019). Possible Contribution of Inflammation-Associated Hypoxia to Increased K2P5.1 K^+^ Channel Expression in CD^4+^ T Cells of the Mouse Model for Inflammatory Bowel Disease. Int. J. Mol. Sci..

[110] Triner D., Xue X., Schwartz A.J., Jung I., Colacino J.A., Shah Y.M. (2017). Epithelial Hypoxia-Inducible Factor 2α Facilitates the Progression of Colon Tumors through Recruiting Neutrophils. Mol. Cell. Biol..

[111] Thompson A.A.R., Elks P.M., Marriott H.M., Eamsamarng S., Higgins K.R., Lewis A., Williams L., Parmar S., Shaw G., McGrath E.E. (2014). Hypoxia-Inducible Factor 2α Regulates Key Neutrophil Functions in Humans, Mice, and Zebrafish. Blood.

[112] Xue X., Ramakrishnan S., Anderson E., Taylor M., Zimmermann E.M., Spence J.R., Huang S., Greenson J.K., Shah Y.M. (2013). Endothelial PAS Domain Protein 1 Activates the Inflammatory Response in the Intestinal Epithelium to Promote Colitis in Mice. Gastroenterology.

[113] Xie L., Xue X., Taylor M., Ramakrishnan S.K., Nagaoka K., Hao C., Gonzalez F.J., Shah Y.M. (2014). Hypoxia-Inducible Factor/MAZ-Dependent Induction of Caveolin-1 Regulates Colon Permeability through Suppression of Occludin, Leading to Hypoxia-Induced Inflammation. Mol. Cell. Biol..

[114] Van Welden S., Laukens D., Ferdinande L., De Vos M., Hindryckx P. (2013). Differential Expression of Prolyl Hydroxylase 1 in Patients with Ulcerative Colitis versus Patients with Crohn’s Disease/Infectious Colitis and Healthy Controls. J. Inflamm..

[115] Tambuwala M.M., Cummins E.P., Lenihan C.R., Kiss J., Stauch M., Scholz C.C., Fraisl P., Lasitschka F., Mollenhauer M., Saunders S.P. (2010). Loss of Prolyl Hydroxylase-1 Protects against Colitis through Reduced Epithelial Cell Apoptosis and Increased Barrier Function. Gastroenterology.

[116] Van Welden S., De Vos M., Wielockx B., Tavernier S.J., Dullaers M., Neyt S., Descamps B., Devisscher L., Devriese S., Van den Bossche L. (2017). Haematopoietic Prolyl Hydroxylase-1 Deficiency Promotes M2 Macrophage Polarization and Is Both Necessary and Sufficient to Protect against Experimental Colitis. J. Pathol..

[117] Chen Y., Zhang H.-S., Fong G.-H., Xi Q.-L., Wu G.-H., Bai C.-G., Ling Z.-Q., Fan L., Xu Y.-M., Qin Y.-Q. (2015). PHD3 Stabilizes the Tight Junction Protein Occludin and Protects Intestinal Epithelial Barrier Function. J. Biol. Chem..

[118] Taniguchi C.M., Miao Y.R., Diep A.N., Wu C., Rankin E.B., Atwood T.F., Xing L., Giaccia A.J. (2014). PHD Inhibition Mitigates and Protects against Radiation-Induced Gastrointestinal Toxicity via HIF2. Sci. Transl. Med..

[119] Tavernier S.J., Vanlangenakker N., Vetters J., Carmeliet P., Janssens S., Lambrecht B.N. (2017). Opposing Regulation and Roles for PHD3 in Lung Dendritic Cells and Alveolar Macrophages. J. Leukoc. Biol..

[120] Chan M.C., Holt-Martyn J.P., Schofield C.J., Ratcliffe P.J. (2016). Pharmacological Targeting of the HIF Hydroxylases—A New Field in Medicine Development. Mol. Asp. Med..

[121] Halligan D.N., Khan M.N., Brown E., Rowan C.R., Coulter I.S., Doherty G.A., Tambuwala M.M., Taylor C.T. (2019). Hypoxia-Inducible Factor Hydroxylase Inhibition Enhances the Protective Effects of Cyclosporine in Colitis. Am. J. Physiol. Gastrointest. Liver Physiol..

[122] Tambuwala M.M., Manresa M.C., Cummins E.P., Aversa V., Coulter I.S., Taylor C.T. (2015). Targeted Delivery of the Hydroxylase Inhibitor DMOG Provides Enhanced Efficacy with Reduced Systemic Exposure in a Murine Model of Colitis. J. Control Release.

[123] Keely S., Campbell E.L., Baird A.W., Hansbro P.M., Shalwitz R.A., Kotsakis A., McNamee E.N., Eltzschig H.K., Kominsky D.J., Colgan S.P. (2014). Contribution of Epithelial Innate Immunity to Systemic Protection Afforded by Prolyl Hydroxylase Inhibition in Murine Colitis. Mucosal Immunol..

[124] Curtis V.F., Ehrentraut S.F., Campbell E.L., Glover L.E., Bayless A., Kelly C.J., Kominsky D.J., Colgan S.P. (2015). Stabilization of HIF through Inhibition of Cullin-2 Neddylation Is Protective in Mucosal Inflammatory Responses. FASEB J..

[125] Gupta R., Chaudhary A.R., Shah B.N., Jadhav A.V., Zambad S.P., Gupta R.C., Deshpande S., Chauthaiwale V., Dutt C. (2014). Therapeutic Treatment with a Novel Hypoxia-Inducible Factor Hydroxylase Inhibitor (TRC160334) Ameliorates Murine Colitis. Clin. Exp. Gastroenterol..

[126] Feng Z., Zou X., Chen Y., Wang H., Duan Y., Bruick R.K. (2018). Modulation of HIF-2α PAS-B Domain Contributes to Physiological Responses. Proc. Natl. Acad. Sci. USA.

[127] Scheuermann T.H., Li Q., Ma H.-W., Key J., Zhang L., Chen R., Garcia J.A., Naidoo J., Longgood J., Frantz D.E. (2013). Allosteric Inhibition of Hypoxia Inducible Factor-2 with Small Molecules. Nat. Chem. Biol..

[128] Ali M.N., Choijookhuu N., Takagi H., Srisowanna N., Nguyen Nhat Huynh M., Yamaguchi Y., Synn Oo P., Tin Htwe Kyaw M., Sato K., Yamaguchi R. (2018). The HDAC Inhibitor, SAHA, Prevents Colonic Inflammation by Suppressing Pro-Inflammatory Cytokines and Chemokines in DSS-Induced Colitis. Acta Histochem. Cytochem..

[129] Cosin-Roger J., Simmen S., Melhem H., Atrott K., Frey-Wagner I., Hausmann M., de Vallière C., Spalinger M.R., Spielmann P., Wenger R.H. (2017). Hypoxia Ameliorates Intestinal Inflammation through NLRP3/MTOR Downregulation and Autophagy Activation. Nat. Commun..

[130] Zhen Y., Zhang H. (2019). NLRP3 Inflammasome and Inflammatory Bowel Disease. Front. Immunol..

[131] Mao L., Kitani A., Similuk M., Oler A.J., Albenberg L., Kelsen J., Aktay A., Quezado M., Yao M., Montgomery-Recht K. (2018). Loss-of-Function CARD8 Mutation Causes NLRP3 Inflammasome Activation and Crohn’s Disease. J. Clin. Investig..

